# Supplementary figures and images for: Novel human monoclonal antibodies with enhanced sensitivity for lipoarabinomannan antigens present in urines of TB patients
Source: bioRxiv. 2026 Jul 27:2026.06.28.735056. Originally published 2026 Jul 1. Preprint. [Version 2] doi: 10.64898/2026.06.28.735056 (PMC13345352; doi:10.64898/2026.06.28.735056)

Suppl. Fig 1


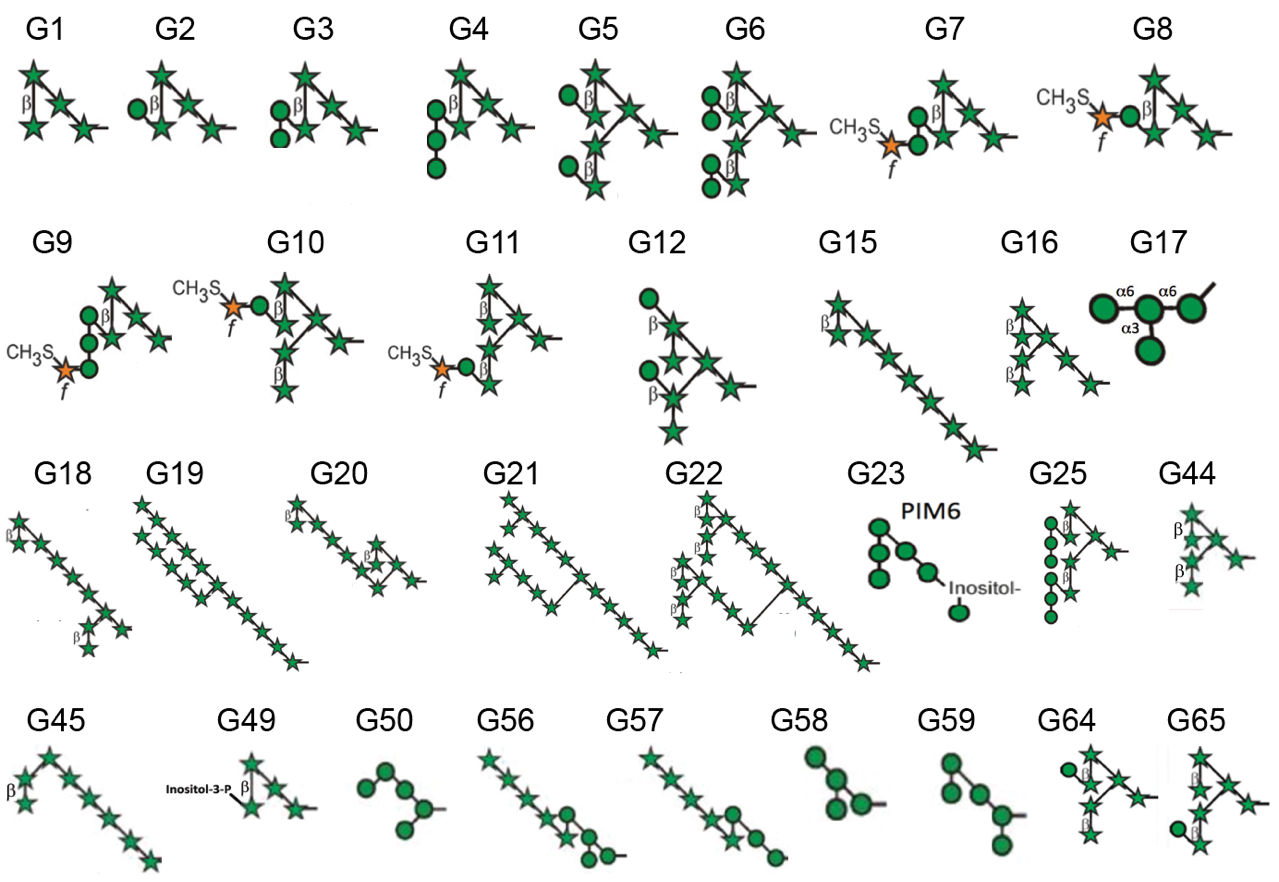
a

b

**Suppl. Fig. 2**

**
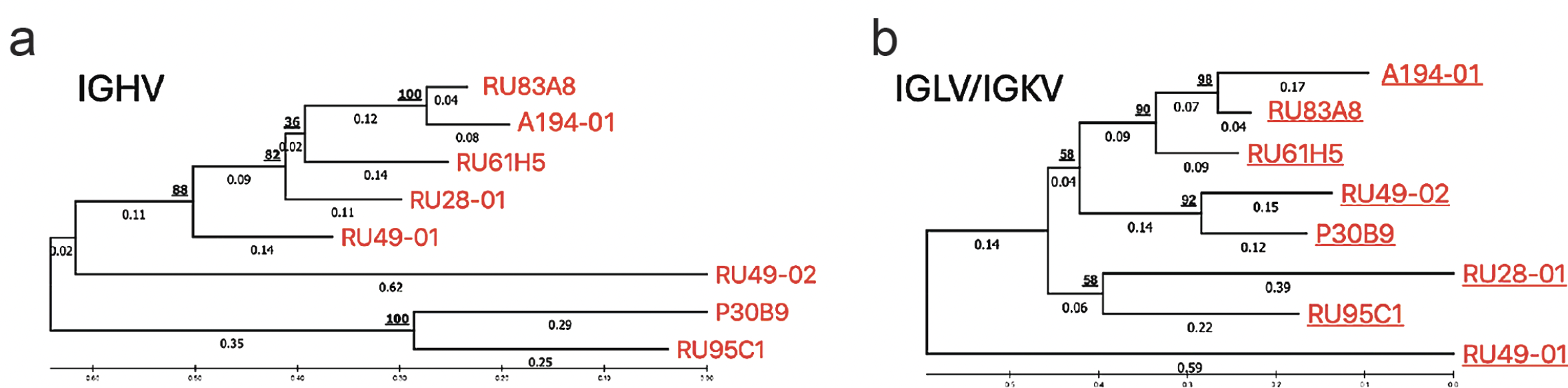
**

.

**Suppl. Fig. 3**

**
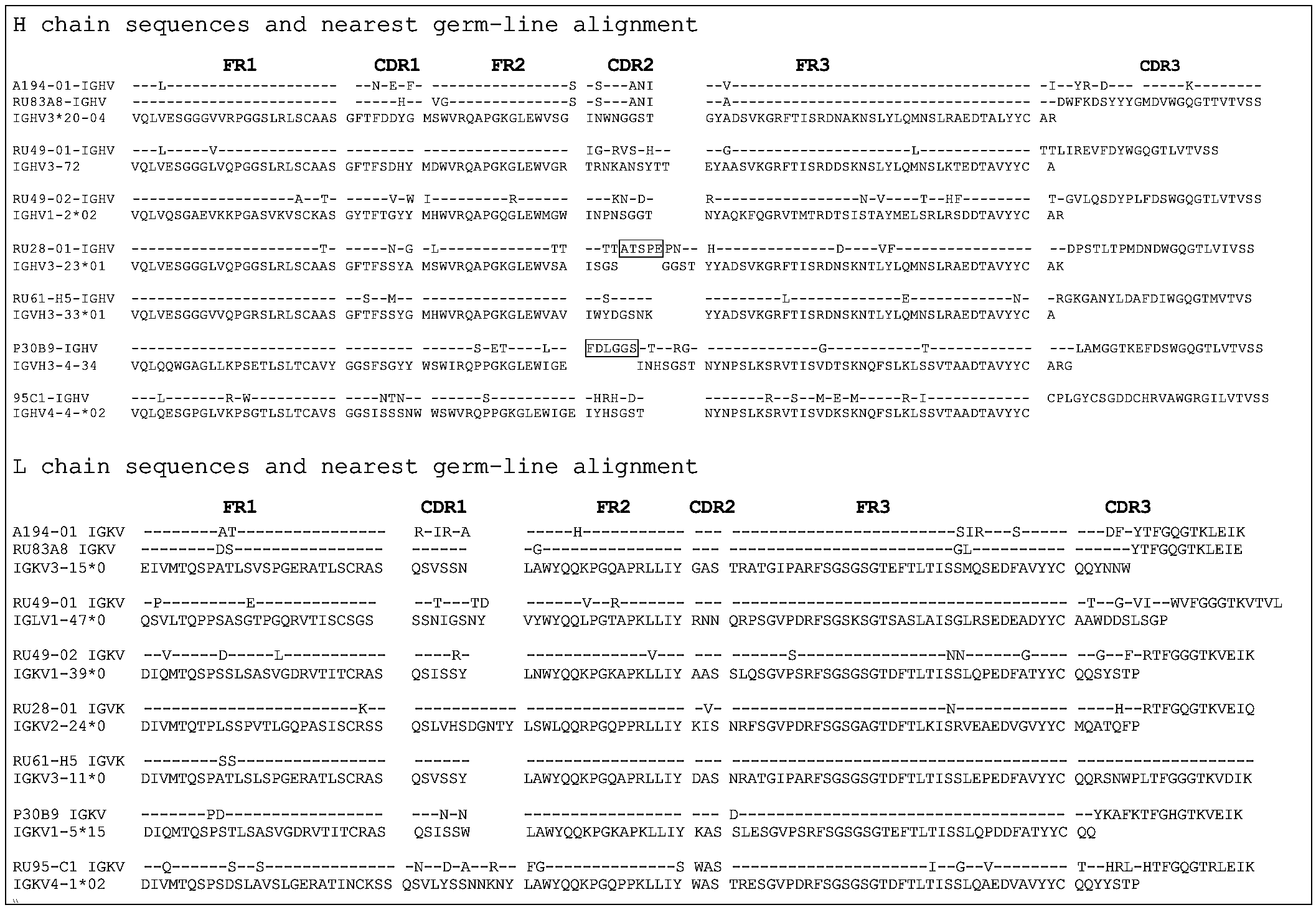
**

**Suppl. Fig. 4**

Supplement: Supplement 1 — Suppl. Fig. 1. Mapping of glycan epitopes recognized by mAbs by binding assays to microarray containing synthetic Ara- and Man-containing glycans representing structural motifs present in ManLAM conjugated to a BSA carrier protein. a- Structures of synthetic glycoconjugates present in the microarray. b- Titration of binding activity of eight mAbs against the synthetic microarray. Suppl. Fig. 2. Phylogenetic analysis of heavy- and light-chain variable region sequences of human anti-LAM monoclonal antibodies. Phylogenetic trees showing sequence relationships among immunoglobulin heavy-chain variable (IGHV; panel ) and light-chain variable (IGLV/IGKV; panel B) regions of human anti-LAM monoclonal antibodies isolated from individuals with M.tb infection. Trees were generated using aligned nucleotide sequences of the variable regions and constructed based on sequence divergence from germline immunoglobulin genes. Branch lengths represent evolutionary distance, and numerical values at branch points indicate bootstrap support values or sequence divergence metrics. Closely clustered antibodies indicate shared sequence similarity and potential clonal relatedness, whereas more distant branches reflect greater diversification of variable region sequences. Suppl. Fig. 3. Alignment of anti-LAM monoclonal antibody variable region sequences with nearest germline genes. Amino acid sequence alignments of immunoglobulin heavy-chain (upper panel) and light-chain (lower panel) variable regions of anti-LAM monoclonal antibodies with their nearest inferred germline sequences are shown. Framework regions (FR1–FR4) and complementarity-determining regions (CDR1–CDR3) are indicated above the alignments according to IMGT numbering. Identical residues relative to the germline sequence are indicated by dashes, whereas amino acid substitutions are shown explicitly. Somatic mutations within framework and CDR regions demonstrate varying levels of affinity maturation among the anti-LAM antibodies. [file media-1.docx]

**Suppl. Table 1.**


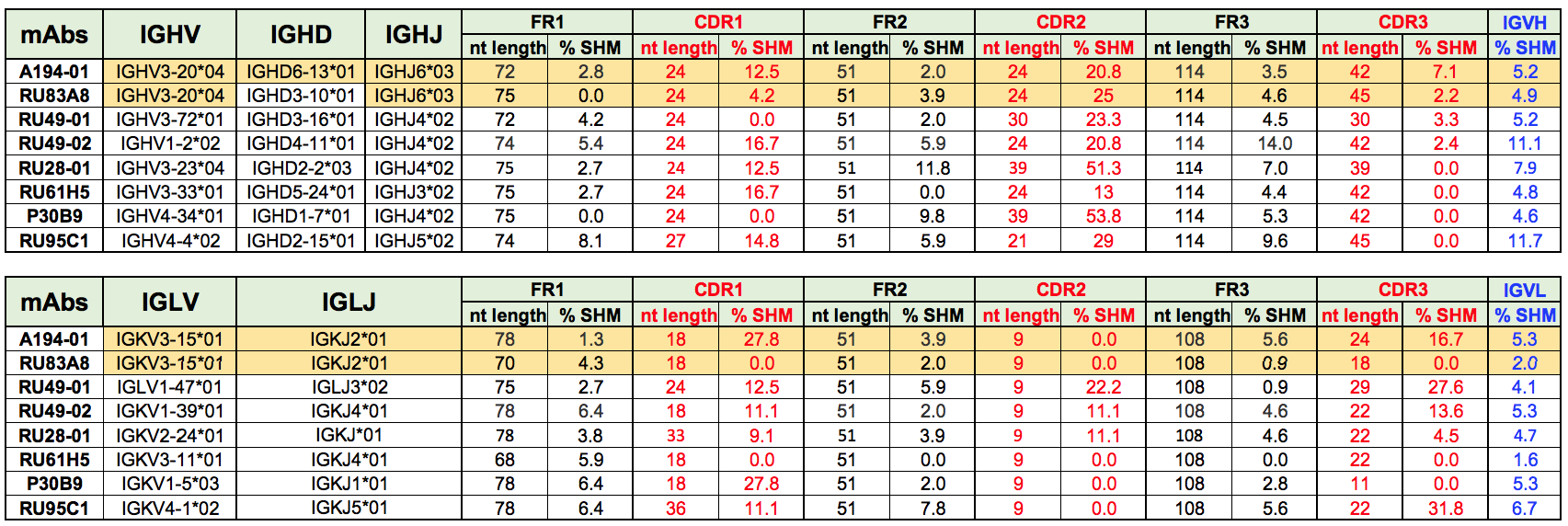

Supplement: Supplement 2 — Suppl.Table 1. Immunoglobulin heavy- and light-chain variable rgion gene usage and somatic hypermutation profiles of human anti-LAM monoclonal antibodies. Summarized are the immunoglobulin variable gene usage, framework region (FR) and complementarity-determining region (CDR) nucleotide lengths, and somatic hypermutation (%SHM) frequencies of the heavy-chain (top panel) and light-chain (bottom panel) variable regions of eight human anti-LAM monoclonal antibodies. Heavy-chain variable (IGHV), diversity (IGHD), and joining (IGHJ) gene segments, as well as light-chain variable (IGKV/IGLV) and joining (IGKJ/IGLJ) gene usage, were assigned by sequence alignment to germline immunoglobulin genes. Nucleotide lengths and percent somatic hypermutation are shown for framework regions (FR1–FR3) and complementarity-determining regions (CDR1–CDR3). Overall variable region somatic hypermutation frequencies for heavy (IGHV) and light (IGLV) chains are indicated in blue. Red text highlights CDR-specific features. [file media-2.docx]
